# Controllability Analysis of Intercellular Protein–Protein Interaction Networks

**DOI:** 10.34133/csbj.0177

**Published:** 2026-08-18

**Authors:** Hikari Nozaki, Keita Tsuchiya, Tatsuya Akutsu, Jose C. Nacher

**Affiliations:** ^1^ Department of Information Science, Faculty of Science, Toho University, Funabashi, Chiba 274-8510, Japan.; ^2^ Bioinformatics Center, Institute for Chemical Research, Kyoto University, Kyoto, Uji 611-0011, Japan.

## Abstract

Understanding how control is organized across interacting cells is an important challenge in computational and systems biology, especially in diseases involving disrupted communication among different cell types. Although network controllability analysis has been widely applied to intracellular biological networks, much less attention has been given to cell–cell interaction networks. A major difficulty is that the underlying protein–protein interaction networks are often undirected, which limits the direct use of maximum-matching-based structural controllability methods. Here, we introduce Minimum Driver Orientation (MDO), a framework that assigns directions to undirected protein–protein interactions so that the number of driver nodes is minimized. We further show theoretically that connected graphs contain no critical nodes under MDO and that quasi-critical nodes constitute a distinct structural control category. We then applied MDO to oligodendrocyte–macrophage (OM) and oligodendrocyte–T-cell (OT) interaction networks relevant to multiple sclerosis (MS). MDO suggested distinct control-node localization patterns in the 2 systems: the OM network showed a more bridge- and macrophage-associated pattern, whereas the OT network showed a more cell-type-specific pattern involving oligodendrocyte and T-cell proteins. Comparisons with random, degree-based, and ligand–receptor-informed orientation schemes showed that the smaller driver-node fractions obtained by MDO were specific to the MDO optimization and were not reproduced by simple orientation rules. Functional annotation and MS-associated protein overlap analyses were interpreted as exploratory biological assessments rather than direct validation of disease mechanisms. Overall, MDO provides a useful computational framework for analyzing maximum-matching-based controllability in undirected intercellular interaction networks.

## Introduction

The integration of control theory with complex networks has led to the development of network controllability, inspiring innovative computational methods and algorithms applied to real-world systems ranging from socio-technical structures to biological pathways. In network controllability, the goal is to identify a set of nodes, called driver nodes, that can steer the entire system to a desired final state in a finite time. Several methods have been proposed, including the maximum matching (MM) and minimum dominating sets (MDS) approaches [1,2]. The feed-back vertex set method has also been introduced to control the system toward specific attractor states [3].

In Liu *et al.* [1], the MM was introduced to identify the driver nodes for the linear dynamical system (*A, B*) described by the equation:

$$\frac{dx\left(t\right)}{\mathrm{dt}}=Ax\left(t\right)+Bu\left(t\right)$$ (1)

where **x**(*t*) is the time-dependent state vector of the *N* nodes, *A* is the weighted directed adjacency matrix of the network, and *B* is the coupling matrix between the external signal **u**(*t*) and the identified driver nodes. In this formulation, a time-dependent input vector **u**(*t*) can drive the state of all nodes to any desired final state within a finite time interval. Driver nodes are identified by constructing a bipartite graph of the original directed graph, and computing a MM. The minimum number of driver nodes is given by *N_D_ =* max{*N − |M|*,1}, where *N* is the total number of nodes in the network, *M* denotes a MM, and |*M*| is the number of matched edges. The unmatched nodes are selected as driver nodes.

The MM approach has led to several applications in biological systems, including the analysis of directed signaling pathways and the identification of disease-associated proteins and drug targets [4]. Compared with MDS, MM-based controllability represents control propagation through directed path structures, allowing upstream root nodes to control distant nodes along the same branch, where the MDS primarily controls nodes through local neighborhood domination. While MDS can be applied to both directed and undirected networks, the MM method was designed for directed systems, limiting its directed applicability to undirected networks. However, in many real-world systems, including socio-technical and biological networks, interactions are often undirected. Therefore, it is desirable to extend the advantages of the MM-based controllability method, such as its ability to implement control propagation along directed paths, to undirected networks.

In biological contexts, most previous studies on network controllability have focused on single-cell networks [4–6], including analyses of metabolic, gene regulatory, and protein–protein interaction (PPI) networks based on MM and MDS frameworks [6–18]. However, advances in multi-omics integration have made it possible to assemble cell–cell interaction networks, opening new opportunities for the large-scale study of intercellular communication [19,20]. In a recent study, cell–cell interaction networks representing oligodendrocyte–macrophage (OM) and oligodendrocyte–T-cell (OT) systems were constructed in the context of multiple sclerosis (MS), revealing several bridging proteins that connect both cellular systems [21]. This represents an important case study for extending the network medicine framework from single-cell to multicellular systems [22].

The resulting networks comprised large-scale PPIs, with some proteins belonging exclusively to one cell type, others expressed in both cell types, and a subset functioning as bridges that mediate ligand–receptor (LR) interactions between the 2 cells. These integrated cell–cell networks are therefore well suited for examining how control-related nodes are distributed across cell-type-specific, shared, and intercellular bridge protein categories. However, because the intracellular interaction layers are based primarily on PPI data, most edges are undirected, preventing the direct application of the MM method.

To address this limitation, we propose a new method called Minimum Driver Orientation (MDO). MDO assigns directions to the edges of an undirected interaction network so that the number of driver nodes is minimized under the MM-based structural controllability framework. We formulate this problem using Integer-Linear Programming (ILP), which optimizes edge orientations for this purpose. Details of the proposed methodology are provided in Materials and Methods. Graph orientation problems have been extensively studied in graph theory and graph algorithms [23,24]. However, to the best of our knowledge, MDO has not previously been studied as a framework for structural controllability in undirected biological networks.

It is well-known that both MM and MDS frameworks generate multiple valid solutions for the same network. To address this issue, nodes are typically categorized as critical, intermittent, or redundant, depending on whether they appear in all, some, or none of the valid control configurations [25,26]. Within the MDO framework, we analyze this problem and obtain 2 key theoretical results. First, there are no critical nodes in a connected graph under MDO. Second, we define a new category, called quasi-critical nodes, which parallels the concept of critical nodes. A node *v_i_* is quasi-critical if it always appears as an endpoint of a path, including an isolated node, in every optimal path–cycle cover. We mathematically prove that a node is quasi-critical if and only if the degree is 0 or 1.

To demonstrate the MDO framework, we analyzed OM and OT networks, both of which have been implicated in neuroinflammatory processes in MS [21]. By applying MDO to orient undirected PPI-based networks, we minimized the number of driver nodes required for MM-based structural controllability and examined how control-related nodes were distributed across cell-type-specific, shared, and bridge protein categories. In the OM network, MDO highlighted a macrophage- and bridge-associated driver-node pattern, whereas in the OT network, MDO showed a more cell-type-specific pattern involving oligodendrocyte and T-cell proteins. This contrasts with the MDS framework, which tended to select control-related nodes from shared or bridge-associated regions of the integrated networks. We also compared MDO with random, degree-based, and LR-informed orientation schemes to evaluate whether the MDO-derived orientations and smaller driver-node fractions were specific to the MDO optimization or could be explained by simpler orientation rules. Finally, we assessed the biological relevance of the identified control-related nodes using GO enrichment analysis and overlap with well-known MS associated proteins. Overall, MDO provides a new framework for analyzing MM-based directional controllability in undirected intercellular interaction networks.

## Materials and Methods

In this study, we propose a new method, called MDO, that enables MM-based controllability analysis of undirected PPI networks. MDO assigns a direction to each edge of an undirected graph, thereby converting it into a directed graph while minimizing the number of driver nodes required for structural controllability. We also derive a theorem and a proposition showing that critical nodes do not exist in connected graphs under MDO, whereas quasi-critical nodes arise as a distinct topological category corresponding to degree-zero or degree-one nodes.

### Data sources

The cell–cell interaction dataset analyzed in this study was obtained from a previously published study [21]. The network was constructed by integrating proteomics data for the analyzed cell types with intracellular and intercellular interaction data from public resources. Additional details on dataset construction are provided in Ref. [21]. MS-related genes were obtained from the Multiple Sclerosis Gene Database (MSGD) [27], a curated resource integrating published information on MS-associated genes and proteins. Because the present analysis was performed on PPI networks, we used the MSGD download file corresponding to “Regulation of protein level”, which is the MSGD category most directly compatible with protein-level network analysis. We then retained human entries and removed duplicate protein entries, resulting in a reference set of 153 human MS-associated proteins. For enrichment analysis, Gene Ontology (GO) libraries were obtained from Enrichr [28], including Cellular Component (2023) (CC), Biological Process (2023) (BP), and Molecular Function (2023) (MF).

### MinDriverOrientation

**Input:** An undirected graph *G*(*V, E*)

**Output:** A directed graph *G*(*V, E_D_*) satisfying the following conditions:

i. Each edge {*u, v*} ∈ *E* is replaced by either (*u, v*) or (*v, u*) (but not both),
ii. The number of driver nodes is the minimum among directed networks satisfying condition (i).

However, such an algorithm is not easy to incorporate various constraints. Hence, we begin with an integer linear program (ILP)-based method.

Let *V* = {*v₁*, …, *v_n_*}. We introduce 0 to 1 variables *x_i,j_* and *x_j,i_* for each edge {*v_i_, v_j_*} ∈ *E*, where we assume without loss of generality that *i* < *j*. Let *X* be the set of 0 to 1 variables *x_i,j_* and *x_j,i_* constructed as above. Then, we use the following ILP to find the MM while orienting each edge:

maximize

$$\sum_{x_{i,j}\in X}x_{i,j}$$ (2)

subject to

$$x_{i,j}+x_{j,i}\leq 1,\;\forall \left\{v_{i},{\;v}_{j}\right\}\in E$$ (3)

$$\sum_{j}\;x_{i,j}\leq 1,\;\forall i\in \left\{1,\ldots ,\;n\right\}$$ (4)

$$\sum_{i}\;x_{i,j}\leq 1,\;\forall j\in \left\{1,\ldots ,\;n\right\}$$ (5)

**Fig. 1.**
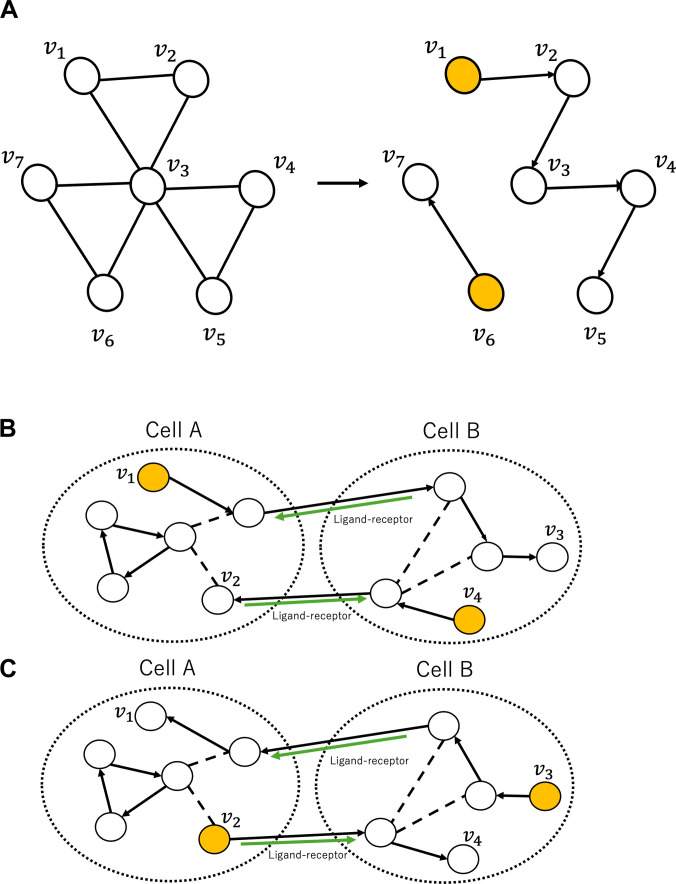
Examples of MinDriverOrientation, path–cycle cover representation, and LR-optimized MDO solution. (A) In this case, $x_{1,2}=x_{2,3}=x_{3,4}=x_{4,5}=x_{6,7}=1,$ and the other $x_{i,j}s$ have value 0. Highlighted nodes $v_{1}$ and $v_{6}$ correspond to the driver nodes. In this case, {$v_{1},v_{3}\}$, {$v_{3},v_{5}\}$, {$v_{3},v_{6}\}$, and {$v_{3},v_{7}\}$ can have any directions. (B) An MDO orientation decomposes the cell–cell interaction network into 2 directed paths and one cycle, shown by solid black arrows. The root of each directed path is assigned as a driver node {$v_{1},v_{4}\}$, shown in yellow, whereas directed cycles do not require driver nodes. Green arrows indicate known ligand–receptor directions. (C) Reorienting paths and cycles can produce an alternative MDO-optimal solution with the same number of driver nodes {$v_{2},v_{3}\}$. Although the total driver-node count is preserved, the identities of driver nodes may change. Such alternative orientations can be used to improve agreement with known ligand–receptor directions and to evaluate the robustness of MDO-derived driver-node assignments. Dashed edges indicate original network edges that are not included in the displayed MDO path–cycle cover, but are retained to show the underlying network structure. In this example, {$v_{1},v_{4},v_{3}\}\;$ are also quasi-critical nodes because they have degree 1.

The meaning of each constraint is as follows (see also Fig. 1A): Each edge {*v_i_, v_j_*} is directed to either (*v*_*i*_, *v_j_*) or (*v*_*j*_, *v_i_*). *x_i,j_* = 1 (resp., *x_j,i_* = 1) means that (*v_i_, v_j_*) (resp., (*v_j_, v_i_*)) is used as a matching edge in the bipartite graph. It is possible that *x_i,j_* = *x_j,i_* = 0 holds for some {*v_i_*, *v_j_*} ∈ *E*. In this case, {*v_i_*, *v_j_*} can have an arbitrary direction [see Eq. (3)]. Each left node in the bipartite graph must be an endpoint of at most one matching edge [see Eq. (4)]. Each right node in the bipartite graph must be an endpoint of at most one matching edge [see Eq. (5)].

Let OPT denote the maximum value of Eq. (1). Finally, the minimum number of driver nodes is given by:

$$\text{max}\left(n-\mathrm{OPT},\;1\right)$$ (6)

and the set of driver nodes is given by:

$$\left\{v_{j}\;|\;\sum_{i}x_{i,j}=0\right\}$$ (7)

(i.e., the set of unmatched right nodes in the bipartite graph).

Although the ILP yields an MDO-optimal orientation, such an orientation is not necessarily unique. In essence, the optimization determines the underlying undirected path–cycle structure that minimizes the number of driver nodes, whereas the directions of individual paths may remain arbitrary. This ambiguity motivates the definition of quasi-critical nodes. Consequently, additional information is needed to determine biologically meaningful path directions. We later assess how the MDO-derived orientations relate to known biological directionality by comparing them with LR data and several baseline orientation strategies. Throughout this manuscript, we use the term “driver nodes” to denote nodes selected as control-input nodes by the corresponding controllability framework. We use the broader term “control-related nodes” when referring collectively to driver nodes and related node categories, such as critical nodes or quasi-critical nodes.

We first define the notion of a path–cycle cover, and then introduce the related concepts of stem, bud, and cactus, which are useful in structural controllability theory and in the proofs of the theorem and proposition derived in this study (see examples in Figs. S1 and S2).

A path–cycle cover of a directed graph is a subgraph containing all nodes of the graph, in which every node belongs to exactly one directed path or one directed cycle. Thus, the graph is decomposed into node-disjoint directed paths and directed cycles that together cover all nodes. An isolated node is regarded as a directed path of length zero. See an example in Fig. 1B and C.

A stem is a simple directed path, that is, a directed path that does not pass through the same node more than once. A path of length zero is also regarded as a stem. A bud is a graph consisting of a simple directed cycle together with one directed edge from an outside node to a node on the cycle. This edge is called the distinguished edge. A cactus is defined recursively as follows: every stem is a cactus, and any graph obtained by attaching a bud to an existing cactus is also a cactus, provided that the bud and the original cactus share only the starting node of the distinguished edge (see Fig. S1 for examples).

Theorem 1. In MDO, there is no critical node if *G*(*V*, *E*) is connected.

**Fig. 2.**
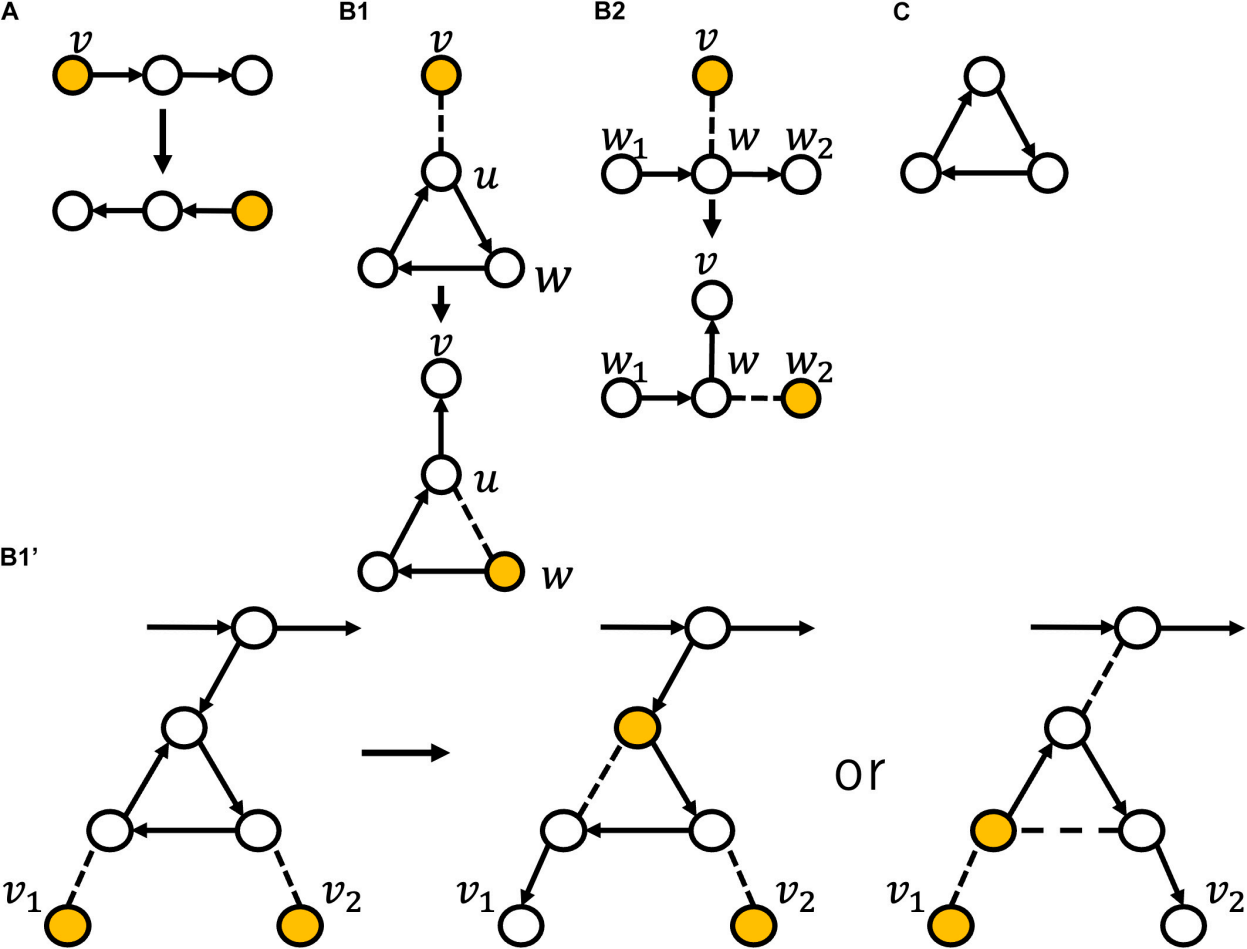
Four cases considered in the proof of Theorem 1. (A) A driver node *v* located at an endpoint of a stem. (B1) An isolated node *v*, represented as a path of length zero, connected to a cycle. (B1ʹ) Multiple driver nodes adjacent to the same bud. (B2) An isolated node *v*, represented as a path of length zero, connected to a stem. (C) A directed cycle in a cactus, where no node can be a driver node. Solid arrows indicated edges in included in an MDO solution, whereas dashed lines represent edges that are not used in the corresponding MDO solution.

**Proof:** Suppose that *v* is a critical node. Then, the following cases can be considered (see also Fig. 2).

1. *v* is an endpoint of a stem (with length at least one) in a cactus. In this case, we reverse the direction of all edges along the stem and let the other endpoint be a driver node (Fig. 2A). Then, all nodes in the original stem are covered by the new stem. Furthermore, all the buds connected to the original stem remain connected to the new stem. Therefore, the nodes in the original cactus are covered by the new cactus.
2. *v* is an isolated node. Since *G* (*V*, *E*) is connected, *v* must be connected to either a cycle (Fig. 2B1 case) or a stem (Fig. 2B2 case). In the former case, let *u* be an arbitrary node in the cycle that is connected to *v* and (*u,w*) be a directed edge in the cycle. Then, we delete (*u,w*), add (*u,v*), and let *w* be a driver node. Then, we have a new stem from *w* to *v*. This new stem covers ***v*** and all nodes in the cycle. In the latter case, suppose that *v* is connected to *w,* and *w* has predecessor node $w_{1}$ and successor node $w_{2}$ in the stem. In this case, we remove a directed edge (*w*, $w_{2}$) and let $w_{2}$ be a driver node. Then, *v* and all nodes in the original stem can be covered by the stem including *w* and the stem beginning from $w_{2}$. If *w* does not have a successor (resp. predecessor) in the original stem, it is enough to add an edge (*w,v*) (resp. (*v,w*)). Note that multiple driver nodes may be adjacent to the same bud as in the Fig. 2B1′ case. In this case, we can move one driver node to another position by converting the bud to a stem.The same argument applies to the case of Fig. 2B2.
3. *v* is in a cycle in the cactus. This case does not occur because any driver node does not have an incoming edge in the cactus (see Fig. 2C).

In any case, we can replace a driver node with another node, without changing the covered nodes. Therefore, there does not exist a critical node. ∎

Next, we introduce the new concept of a quasi-critical node. A node *v_i_* is called a quasi-critical node if it always appears as an endpoint of a path, including an isolated node, in every optimal path–cycle cover. A node *v_i_* is called a redundant node if it never appears as an endpoint of a path, including an isolated node, in any optimal path–cycle cover. In all other cases, *v_i_* is called an intermittent node. In what follows, we demonstrate the fundamental structural property of quasi-critical nodes (see Fig. 1B and C and Fig. S2).

Proposition 1. *v_i_* is quasi-critical if and only if the degree of *v_i_* is 0 or 1.

**Fig. 3.**
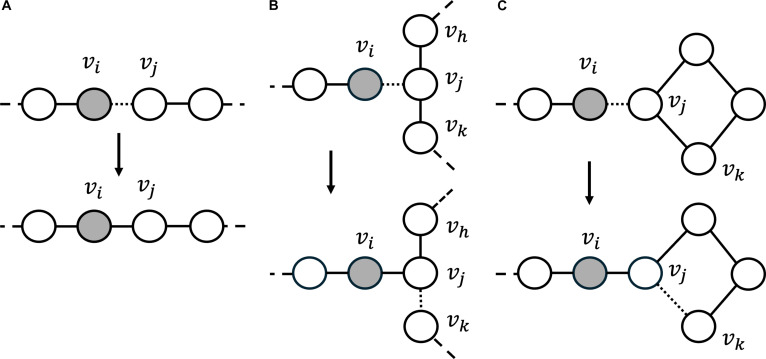
Three cases considered in the proof of Proposition 1. *(A) v_i_* is adjacent to an endpoint *v_j_* of another path. (B) *v_i_* is adjacent to an internal node *v_j_* of another path. (C) *v_i_* is adjacent to a node *v_j_* in a cycle. Bold solid lines and bold dashed lines represent edges and portions of paths in a path–cycle cover, respectively. Thin dotted lines represent edges that are not included in the path–cycle cover.

**Proof:** It is obvious for the case of degree 0. Furthermore, the “if” part is obvious. Therefore, we prove the “only if” part for the case of degree 1. Suppose that $v_{i}$ has degree at least 2 and is an endpoint of a path in an optimal path–cycle cover *C*. By considering the following 3 possible cases (see also Fig. 3), the proposition holds.

1. $v_{i}$ has a neighboring node $v_{j}$ that is an endpoint of another path in *C* (Fig. 3A). We add an edge {$v_{i},v_{j}$} to *C.* Then, the resulting set of edges is also a path–cycle cover. Furthermore, since 2 paths are merged, the number of paths is smaller than that for *C*, which contradicts the assumption that *C* is optimal. Therefore, this case cannot occur.

2. $v_{i}\;$ has a neighboring node $v_{j}\;$ that is a node (but not an endpoint) of another path in *C* (Fig. 3B). Let $v_{h}$ and $v_{k}$ be neighboring nodes of $v_{j}\;$ in that path. We remove {$v_{j},v_{k}$} from *C* and add {$v_{i},v_{j}$} to *C*. Then, the resulting set of edges is also a path–cycle cover with keeping the number of paths in the cover. Furthermore, $v_{i}$ is no longer an endpoint of the path, which means that $v_{i}$ is an intermittent node.

3. $v_{i}$ has a neighboring node $v_{j}$ that is a node of a cycle in *C* (Fig. 3C). Let $v_{k}$ be a neighboring node of $v_{j}\;$ in the cycle*.* We remove {$v_{j},v_{k}$} from *C* and add {$v_{i},v_{j}$} to *C*. Then, the resulting set of edges is also a path–cycle cover with keeping the number of paths in the cover. Furthermore, $v_{i}$ is no longer an endpoint of the path, which again means that *v_i_* is an intermittent node. ∎

### Evaluation of LR direction agreement and orientation baselines

To assess whether the orientations assigned by MDO were biologically consistent with known intercellular signaling directions, we compared assigned edge directions with known LR directions. Only strictly directional LR interactions were used for this evaluation as shown in our dataset [21]. Bidirectional LR pairs were excluded from the direction-agreement calculation. For each orientation method, LR direction agreement was defined as the fraction of LR edges whose MDO-based assigned direction matched the known LR direction. We compared MDO with the following battery of 5 orientation baselines. First, in the random-orientation baseline, each undirected edge was assigned 1 of the 2 possible directions uniformly at random. In the degree-based baselines, each edge was oriented either from the lower-degree (L) node to the higher-degree (H) node or from the higher-degree (H) node to the lower-degree (L) node, where degrees were computed in the original undirected network. When the 2 endpoints had the same degree, the edge was removed from network. For random-orientation baseline, 100 oriented networks were generated. The number of driver nodes in each of 3 edge orientation baselines was computed by MM [1]. We also considered 2 LR-informed MDO analyses. First, in the LR-optimized MDO reorientation analysis, known LR directions were used as a secondary criterion to select among MDO-optimal path–cycle orientations without changing the minimum driver-node count (see Fig. 1B and C). This analysis was used to evaluate whether alternative MDO-optimal orientations could improve agreement with known LR directions without changing the minimum driver-node count, and whether the identities of driver nodes and quasi-critical nodes were robust under such reorientation. Secondly, in the LR-constrained MDO analysis, known strictly directional LR edges were imposed as hard constraints in the ILP formulation, and the MDO problem was solved again. This analysis was used to evaluate whether biologically established LR directionality is compatible with near-optimal structural controllability.

### Enrichment analysis

The overrepresentation of each protein category among control-related node sets was quantified using a fraction-based enrichment ratio, defined as the fraction of nodes from that category within the control-related node set divided by the corresponding fraction in the whole network. Statistical significance of category overrepresentation (enrichment) was assessed using one-sided Fisher’s exact tests.

GO enrichment analysis was performed using the Enrichr platform [28]. GO enrichment analyses were performed separately for the 3 GO domains: BP, MF, and CC. For each GO term, nominal Fisher-based *P* values and odds ratios were obtained from Enrichr.

To provide an additional assessment based on random node-set sampling within each network, empirical *P* values (*P*_emp_) were computed under a network-aware null model. Specifically, let *D* denote the set of observed control-related node set with |*D*| = *k* and let *V* denote the set of all network nodes. For each GO term, we first computed the observed Fisher’s exact test *P* value *P*_obs_. We then generated *n*_perm_ = 50,000 random subsets *R_i_ ⊂ V* such that |*R_i_*| = *k*, and recomputed the corresponding Fisher’s exact test *P* values *P*_rand_^(^*^i^*^)^. The empirical *P* value was defined as the proportion of random samples yielding a *P* value less than or equal to the observed one, using

$$P_{\mathrm{emp}}=\left(1+s\right)/\left(1+n_{\text{perm}}\right)$$ (8)

where *s* is the number of random samples satisfying *P*_rand_^(*i*)^ ≤ *P*_obs*.*_ This formulation avoids zero empirical *P* values when no random sample is more significant than the observed result.

Multiple-testing correction for GO enrichment was performed using the Benjamini–Hochberg (BH) procedure. Nominal *P* values, empirical *P* values, and BH-corrected *q* values were reported in Tables 1 and 2, as well as in Table S1. GO terms with BH-corrected (*q* < 0.10) were considered significant after multiple-testing correction, whereas terms with nominal (*P* < 0.05) or (*P*_emp_ < 0.05) but (*q* ≥ 0.10) were interpreted as exploratory enrichment patterns.

**Table 1.** Representative Gene Ontology enrichment results for control-node sets in the OM network. Nominal *P* values from Fisher’s exact test, empirical permutation *P* values (*P*_emp_; 50,000 permutations), and BH-corrected *q* values are shown. Terms with *q* < 0.10 were considered significant after multiple-testing correction, whereas nominally enriched terms with *q* ≥ 0.10 were treated as exploratory. The number of overlapping proteins indicates the overlap between each control-related node set and the corresponding GO term. Full results, including LR-optimized MDO, LR-constrained MDO, and quasi-critical/critical node sets, are provided in Table S1 (Excel file).

Method	Control node set	GO	Representative GO term	Overlap	*P* value	*P*_emp_ value	*q* value	Interpretation
MDO	Driver	BP	Fibrinolysis (GO:0042730)	6	6.31 × 10^−4^	4.60 × 10^−4^	7.42 × 10^−1^	Exploratory
MDO	Driver	BP	Diacylglycerol metabolic process (GO:0046339)	4	9.98 × 10^−4^	9.20 × 10^−4^	7.42 × 10^−1^	Exploratory
MDO	Driver	CC	T cell receptor complex (GO:0042101)	5	1.18 × 10^−2^	3.38 × 10^−3^	1.0	Exploratory
MDO	Driver	CC	Platelet dense granule (GO:0042827)	5	2.21 × 10^−2^	7.64 × 10^−3^	1.0	Exploratory
MDO	Driver	MF	Carbon-nitrogen ligase activity, with glutamine as amido-N-donor (GO:0016884)	3	9.68 × 10^−3^	3.68 × 10^−3^	1.0	Exploratory
MDO	Driver	MF	Hydrolase activity, hydrolyzing N-glycosyl compounds (GO:0016799)	5	1.37 × 10^−2^	3.84 × 10^−3^	1.0	Exploratory
MDS	Driver	BP	Positive regulation of intracellular signal transduction (GO:1902533)	52	6.05 × 10^−8^	2.00 × 10^−5^	1.99 × 10^−4^	FDR-significant
MDS	Driver	BP	Positive regulation of protein phosphorylation (GO:0001934)	41	3.92 × 10^−7^	2.00 × 10^−5^	5.44 × 10^−4^	FDR-significant
MDS	Driver	CC	Membrane raft (GO:0045121)	22	4.85 × 10^−3^	4.96 × 10^−3^	1.0	Exploratory
MDS	Driver	CC	Dendrite (GO:0030425)	20	1.19 × 10^−2^	1.16 × 10^−2^	1.0	Exploratory
MDS	Driver	MF	MHC class II protein complex binding (GO:0023026)	10	1.25 × 10^−3^	6.00 × 10^−4^	3.03 × 10^−1^	Exploratory
MDS	Driver	MF	Protein kinase binding (GO:0019901)	42	1.35 × 10^−3^	7.20 × 10^−4^	3.03 × 10^−1^	Exploratory
MDS	Critical	BP	Positive regulation of intracellular signal transduction (GO:1902533)	29	3.75 × 10^−8^	2.00 × 10^−5^	8.02 × 10^−5^	FDR-significant
MDS	Critical	BP	Regulation of apoptotic process (GO:0042981)	34	8.75 × 10^−8^	2.00 × 10^−5^	9.36 × 10^−5^	FDR-significant
MDS	Critical	CC	Focal adhesion (GO:0005925)	30	2.00 × 10^−4^	1.80 × 10^−4^	2.08 × 10^−2^	FDR-significant
MDS	Critical	CC	Cell-substrate junction (GO:0030055)	30	2.31 × 10^−4^	1.80 × 10^−4^	2.08 × 10^−2^	FDR-significant
MDS	Critical	MF	Protein kinase binding (GO:0019901)	23	1.71 × 10^−4^	6.00 × 10^−5^	2.72 × 10^−2^	FDR-significant
MDS	Critical	MF	MHC class II protein complex binding (GO:0023026)	7	2.62 × 10^−4^	4.00 × 10^−5^	2.72 × 10^−2^	FDR-significant

**Table 2.** Representative Gene Ontology enrichment results for control-node sets in the OT network. Nominal *P* values from Fisher’s exact test, empirical permutation values (*P*_emp_; 50,000 permutations), and BH-corrected *q* values are shown. Terms with *q* < 0.10 were considered significant after multiple-testing correction, whereas nominally enriched terms with *q* ≥ 0.10 were treated as exploratory. The number of overlapping proteins indicates the overlap between each control-related node set and the corresponding GO term. Full results, including LR-optimized MDO, LR-constrained MDO, and quasi-critical/critical node sets, are provided in Table S1 (Excel file).

Method	Control node set	GO	Representative GO term	Overlap	*P* value	*P*_emp_ value	*q* value	Interpretation
MDO	Driver	BP	Nucleotide-sugar metabolic process (GO:0009225)	5	1.20 × 10^−3^	5.00 × 10^−4^	1.0	Exploratory
MDO	Driver	BP	Inorganic cation import across plasma membrane (GO:0098659)	8	1.44 × 10^−3^	3.60 × 10^−4^	1.0	Exploratory
MDO	Driver	CC	Excitatory synapse (GO:0060076)	3	4.96 × 10^−3^	1.88 × 10^−3^	1.0	Exploratory
MDO	Driver	CC	GABA-ergic synapse (GO:0098982)	2	6.31 × 10^−2^	3.34 × 10^−2^	1.0	Exploratory
MDO	Driver	MF	Potassium:Chloride symporter activity (GO:0015379)	3	5.42 × 10^−3^	1.90 × 10^−3^	9.71 × 10^−1^	Exploratory
MDO	Driver	MF	Chloride transmembrane transporter activity (GO:0015108)	6	1.20 × 10^−2^	1.08 × 10^−2^	9.71 × 10^−1^	Exploratory
MDS	Driver	BP	Regulation of ERK1 and ERK2 cascade (GO:0070372)	27	4.32 × 10^−6^	2.00 × 10^−5^	1.55 × 10^−2^	FDR-significant
MDS	Driver	BP	Muscle cell development (GO:0055001)	7	2.01 × 10^−5^	6.00 × 10^−5^	3.09 × 10^−2^	FDR-significant
MDS	Driver	CC	Focal adhesion (GO:0005925)	57	2.77 × 10^−3^	2.36 × 10^−3^	3.32 × 10^−1^	Exploratory
MDS	Driver	CC	Secretory granule lumen (GO:0034774)	48	2.82 × 10^−3^	2.60 × 10^−3^	3.32 × 10^−1^	Exploratory
MDS	Driver	MF	Ubiquitin protein ligase binding (GO:0031625)	47	9.41 × 10^−6^	2.00 × 10^−5^	5.37 × 10^−3^	FDR-significant
MDS	Driver	MF	Ubiquitin-like protein ligase binding (GO:0044389)	48	2.56 × 10^−5^	4.00 × 10^−5^	7.01 × 10^−3^	FDR-significant
MDS	Critical	BP	ERBB2 signaling pathway (GO:0038128)	4	1.74 × 10^−5^	2.00 × 10^−5^	3.01 × 10^−2^	FDR-significant
MDS	Critical	BP	Regulation of protein phosphorylation (GO:0001932)	18	3.87 × 10^−5^	6.00 × 10^−5^	3.01 × 10^−2^	FDR-significant
MDS	Critical	CC	Golgi-associated vesicle (GO:0005798)	6	2.16 × 10^−3^	3.40 × 10^−4^	2.38 × 10^−1^	Exploratory
MDS	Critical	CC	Focal adhesion (GO:0005925)	27	3.11 × 10^−3^	2.52 × 10^−3^	2.38 × 10^−1^	Exploratory
MDS	Critical	MF	Ephrin receptor binding (GO:0046875)	7	7.27 × 10^−7^	2.00 × 10^−5^	2.86 × 10^−4^	FDR-significant
MDS	Critical	MF	Ubiquitin-like protein ligase binding (GO:0044389)	24	9.44 × 10^−5^	4.00 × 10^−5^	1.25 × 10^−2^	FDR-significant

## Results

The theoretical properties of the proposed MDO computational method, including mathematical results such as Theorem 1 and Proposition 1, are described in Materials and Methods. The following sections focus on the computational results obtained by applying the proposed controllability framework to cell–cell interaction networks.

### MDO identifies a smaller driver node fraction in the larger OT network

Using the cell–cell interaction network data for the OM and OT systems, we analyzed the fraction of nodes required for control under 2 frameworks: MDO and MDS. From the original data, we extracted the largest connected component for each network. The OM network was composed of 3,562 nodes, and 33,326 edges, with an average degree of <*k*> = 18.71, whereas the OT network was larger, consisting of 8,150 nodes and 105,351 edges, with an average degree <*k*> = 25.85.

**Fig. 4.**
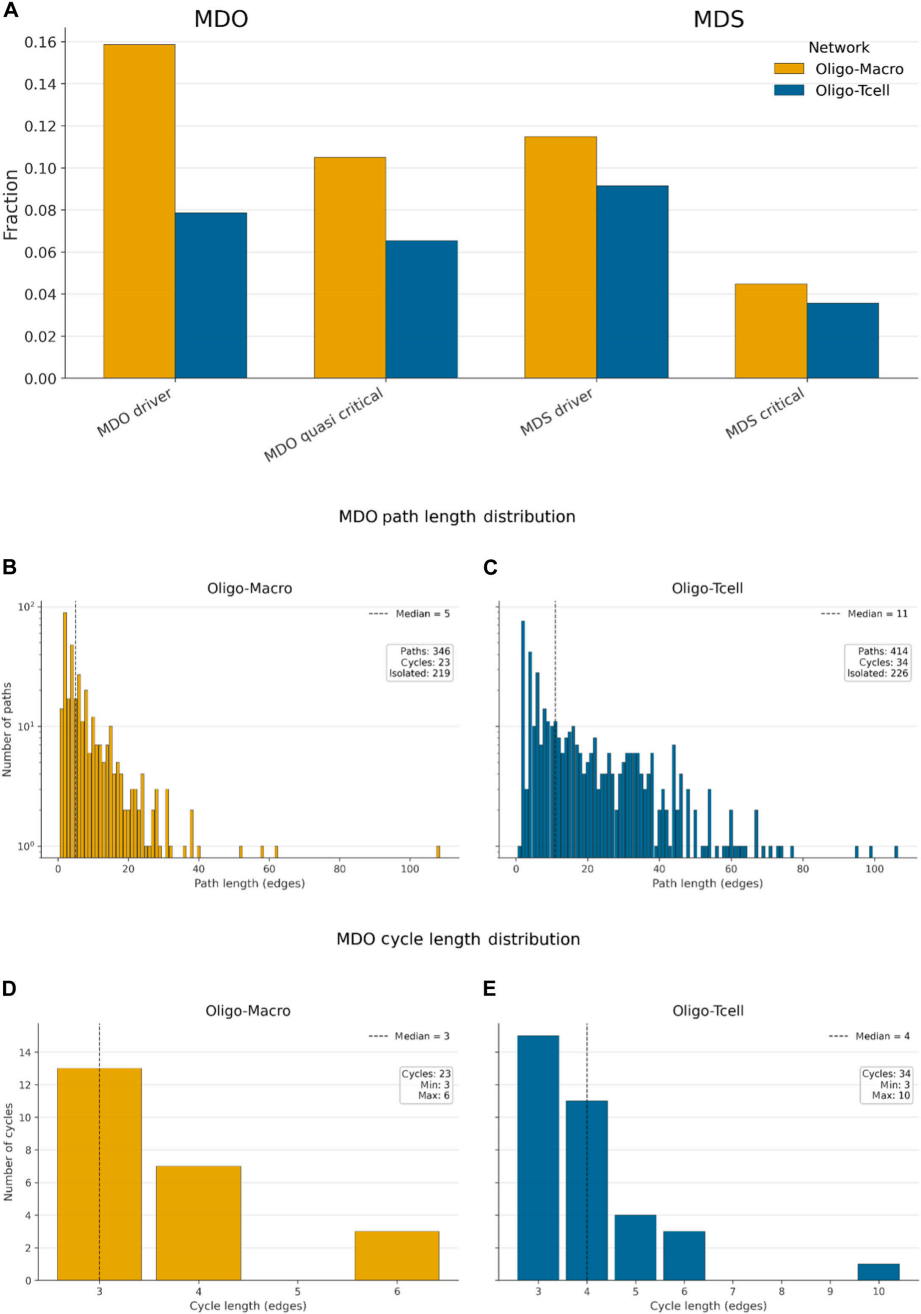
Driver-node fractions and MDO-induced path–cycle cover structures in the OM and OT cell–cell interaction networks. (A) Fractions of control-related nodes identified in the entire OM and OT networks. Bars show the proportions of MDO driver nodes, MDO quasi-critical nodes, MDS driver, and MDS critical nodes. MDO identified a smaller fraction of driver and quasi-critical nodes in the OT than in the OM network. (B to E) Path- and cycle-length distributions in the MDO-induced path–cycle covers of the OM and OT networks. Panels (B) and (C) show directed path-length distributions for the OM and OT networks, respectively, whereas panels (D) and (E) show directed cycle-length distributions for the OM and OT networks, respectively. The OT network showed broader path- and cycle-length distributions, indicating that more nodes are covered by longer paths and cycles under the MDO solution. In particular, the longer MDO-induced paths in OT are consistent with the smaller fraction of driver nodes required in this network.

Figure 4 compares the fractions of driver, quasi-critical, and critical nodes identified by the MDO and MDS models in both networks. In the MDO model, the fractions of driver nodes and quasi-critical nodes in the OT network were considerably smaller than those in the OM network (7.8% and 6.5% in OT, compared with 15.8% and 10.5% in OM, respectively).

Interestingly, although the OT network contained far more nodes and edges, its relative proportion of driver nodes was lower. The OM and OT networks had similar global graph-distance properties, with average shortest path lengths of *L* = 3.11 and *L* = 3.05, and diameters of *d* = 10 and *d* = 8, respectively. However, because global graph-distance measures such as average shortest path length and diameter do not directly determine the number of MDO driver nodes, we further examined the MDO-induced path–cycle cover structure (Fig. 4). In the MDO framework, each directed path requires one driver node at its root, whereas directed cycles do not require driver nodes. Isolated nodes are treated as paths of length zero and therefore also require driver nodes. The OM network contained 346 nonisolated paths and 219 isolated nodes, corresponding to 565 path roots among 3,562 nodes. In contrast, the OT network contained 414 nonisolated paths and 226 isolated nodes, corresponding to 640 path roots among 8,150 nodes (see Fig. 4). Thus, although the OT network had a larger absolute number of paths, the number of path roots relative to network size was much smaller in OT than in OM. Consistently, the median path length was longer in OT than in OM (11 edges vs. 5 edges), indicating that each driver node covers a longer directed path on average. This provides a more direct explanation for why the larger OT network shows a lower driver-node fraction under the MDO framework.

When comparing control models, MDS selected a smaller fraction of driver nodes than MDO in the OM network, whereas MDO required fewer driver nodes than MDS in the OT network (Fig. 4A). These differences should be interpreted cautiously, because MDO and MDS represent different notions of network control: MDO is based on MM structural controllability and optimized directed path–cycle covers, allowing control to propagate along directed path structures (see Fig. 4B to E), whereas MDS is based on domination in an undirected local-neighborhood structure. Therefore, the comparison should not be interpreted as identifying a universally superior model. Despite these model-specific differences, both MDO and MDS identified relatively small subsets of control-related nodes in both networks, suggesting that each framework captures a complementary and compact driver-node organization from a different theoretical perspective. For the MDO model specifically, the lower driver-node fraction in OT can be better understood from the MDO-induced path–cycle cover structure

### Evaluation of MDO driver nodes and orientations using LR directionality and baseline orientation rules

**Fig. 5.**
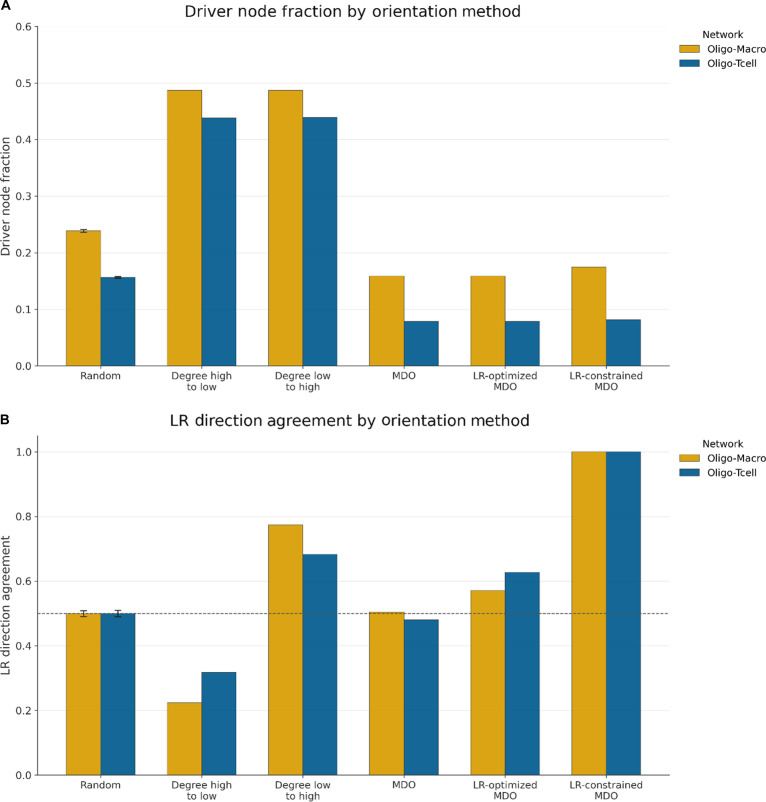
Comparison of driver-node fraction and ligand–receptor direction agreement across orientation methods. (A) Fraction of driver nodes obtained for the OM and OT networks under MDO, random orientation, degree-based orientation baselines, LR-optimized MDO, and LR-constrained MDO. Bars show OM in yellow and OT in blue. For the random-orientation baseline, bars and error bars represent the mean ± standard deviation over 100 random trials. (B) Agreement between assigned edge directions and known strictly directional ligand–receptor directions for the same orientation methods. Random orientation showed approximately 50% agreement, whereas LR-constrained MDO showed 100% agreement by construction because known LR directions were imposed as hard constraints. OM, oligodendrocyte–macrophage; OT, oligodendrocyte–T cell.

To evaluate whether the MDO-derived orientations and the smaller driver-node fractions were specific to the MDO optimization or could be reproduced by simpler orientation rules, we compared unconstrained MDO with random and degree-based orientation baselines, as well as 2 LR-based MDO variants (see Fig. 5) as described in Materials and Methods. Notably, the quasi-critical node set was identical across the explored MDO baselines and LR-based variants MDO. This follows from Proposition 1, which shows that quasi-critical nodes correspond to degree-one nodes in the underlying network. Because the degree-based orientation baselines and LR-based variants do not alter the underlying undirected topology, the quasi-critical node set remains unchanged. First, for the random-orientation baseline, the average driver-node fraction was 23.8% in OM and 15.6% in OT. Degree-based orientations required substantially larger driver-node fractions, with high-degree-to-low-degree and low-degree-to-high-degree orientations both requiring approximately 48.7% of nodes in OM and 43.8% of nodes in OT. In contrast, unconstrained MDO required only 15.8% driver nodes in OM and 7.8% in OT. Thus, MDO produced a markedly smaller driver-node fraction than random or degree-based orientation strategies, indicating that the reduction in driver nodes is specific to the MDO optimization and is not reproduced by simple orientation heuristics.

We next evaluated agreement with known strictly directional LR interactions. As expected, random orientation showed approximately 50% agreement. Unconstrained MDO showed agreement close to the random baseline, with 50.5% in OM and 48% in OT, indicating that MDO alone should not be interpreted as a direct predictor of LR directionality. LR-optimized MDO reorientation improved the agreement to 57% in OM and 62.7% in OT without changing the minimum driver-node count (565 and 640 driver nodes in OM and OT networks, respectively). Interestingly, the low-degree-to-high-degree baseline showed higher LR agreement, reaching 77% in OM and 68% in OT. This suggests that LR directionality in these cell–cell networks is partially degree-biased, possibly reflecting the tendency of ligand nodes to be more specialized and receptor-associated nodes to be more highly connected. However, this degree-based rule required many more driver nodes than MDO (1,735 and 3,578 driver nodes for OM and OT networks, respectively), indicating that it captures a local LR-direction tendency but does not reproduce the global controllability optimization achieved by MDO (see Fig. 5A). For the high-degree-to-high-degree baseline, the LR agreement decreases below 50%, namely, 22% for the OM network and 31% for the OT network (see Fig. 5 and Table S3 for numerical values).

In addition, we performed LR-constrained MDO, in which known strictly directional LR edges were imposed as hard constraints in the ILP formulation. By construction, this analysis gives 100% agreement with the fixed LR directions, and therefore, it should not be interpreted as LR-direction prediction. Instead, it evaluates how imposing known biological LR directionality affects the MDO controllability solution. The driver-node fraction increased from 15.8% to 17.5% in OM and from 7.8% to 8.2% in OT, indicating that enforcing known LR directions changed the MDO solution while keeping the driver-node fraction close to that of unconstrained MDO (see Fig. 5A). However, because LR-direction agreement is imposed by construction in this analysis, it should not be regarded as an independent validation of orientation recovery. Moreover, because the identities of selected driver nodes and the corresponding enrichment results can change under the LR-constrained solution, we treat LR-constrained MDO as a sensitivity analysis rather than as a replacement for the unconstrained or LR-optimized MDO models. For completeness, the enrichment results for LR-constrained MDO are also provided in figures and in supplementary tables.

### Localization of control-related nodes across cell-category protein groups

**Fig. 6.**
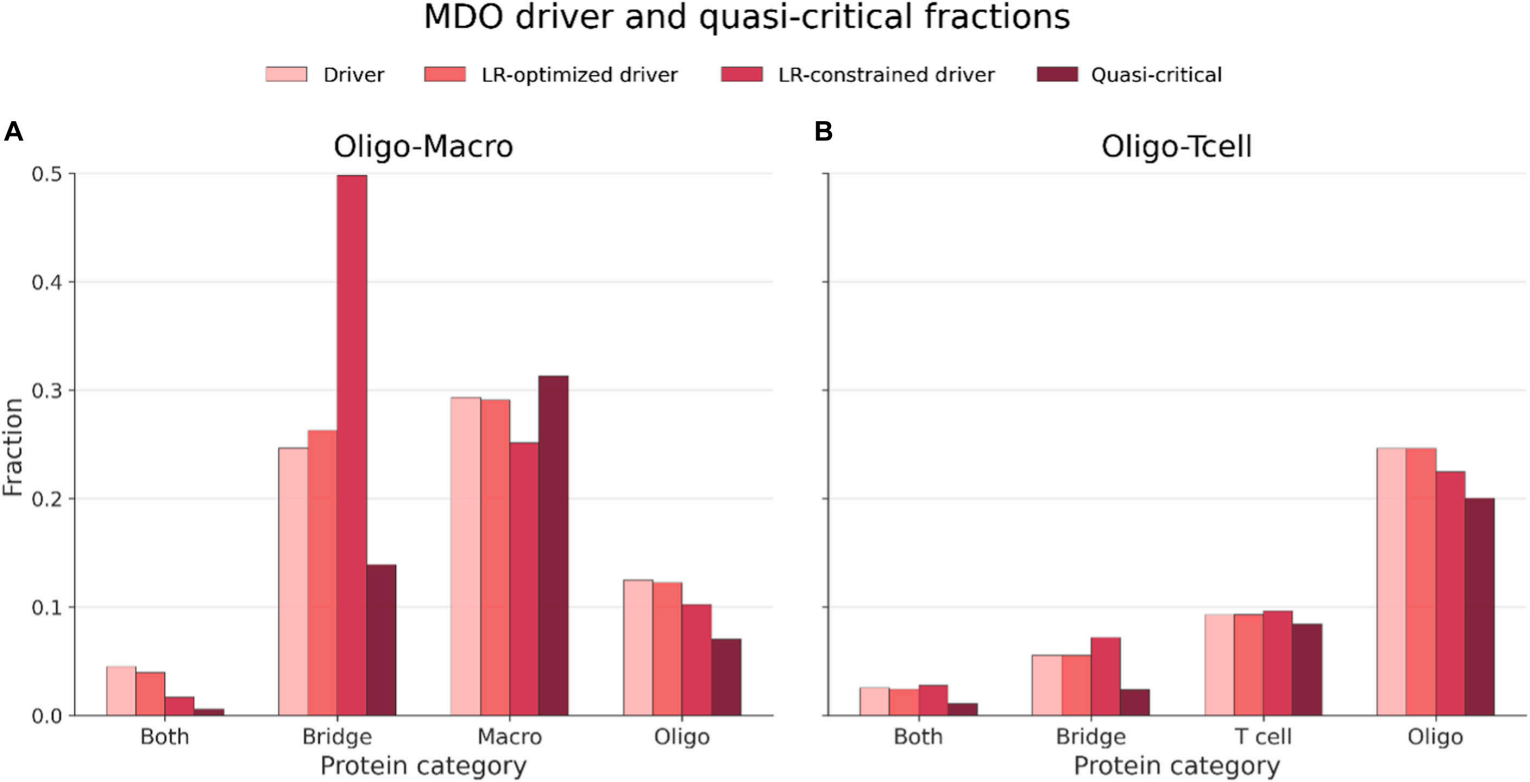
Fraction of MDO driver and quasi-critical nodes across protein categories. Fractions of category nodes identified as unconstrained MDO drivers, LR-optimized MDO drivers, LR-constrained MDO drivers, or quasi-critical nodes are shown for the (A) OM and (B) OT networks. Protein categories were defined by cell-type assignment: both-cell proteins, bridge proteins mediating ligand–receptor interactions, and cell-type-specific proteins.

We next examined the localization of control-related nodes across protein categories defined by cell-type assignment. Proteins were classified as cell-type-specific proteins, proteins expressed in both cell types, or bridge proteins mediating LR interactions between the 2 cell types. For the MDO model, we compared unconstrained MDO driver nodes, LR-optimized MDO driver nodes, LR-constrained MDO driver nodes, and quasi-critical nodes (Fig. 6). In the OM network, unconstrained MDO and LR-optimized MDO showed similar localization profiles, with relatively high fractions of driver nodes in the bridge and macrophage protein categories. Quasi-critical nodes were also most prominent in the macrophage category, followed by the bridge category. Under LR-constrained MDO, the localization of driver nodes shifted more strongly toward the bridge category, indicating that imposing known LR directions can affect driver-node identities and their distribution across protein categories.

In the OT network, MDO driver nodes were mainly localized in the oligodendrocyte and T-cell protein categories, with the highest fraction observed in the oligodendrocyte category. This pattern was largely preserved under LR-optimized MDO and was only moderately altered under LR-constrained MDO. Quasi-critical nodes also showed higher fractions in the oligodendrocyte and T-cell categories than in the bridge or both-cell categories. These results suggest that, under MDO, the OM network has a more bridge/macrophage-associated control-node localization, whereas the OT network shows a more cell-type-specific localization involving both oligodendrocyte and T-cell proteins.

**Fig. 7.**
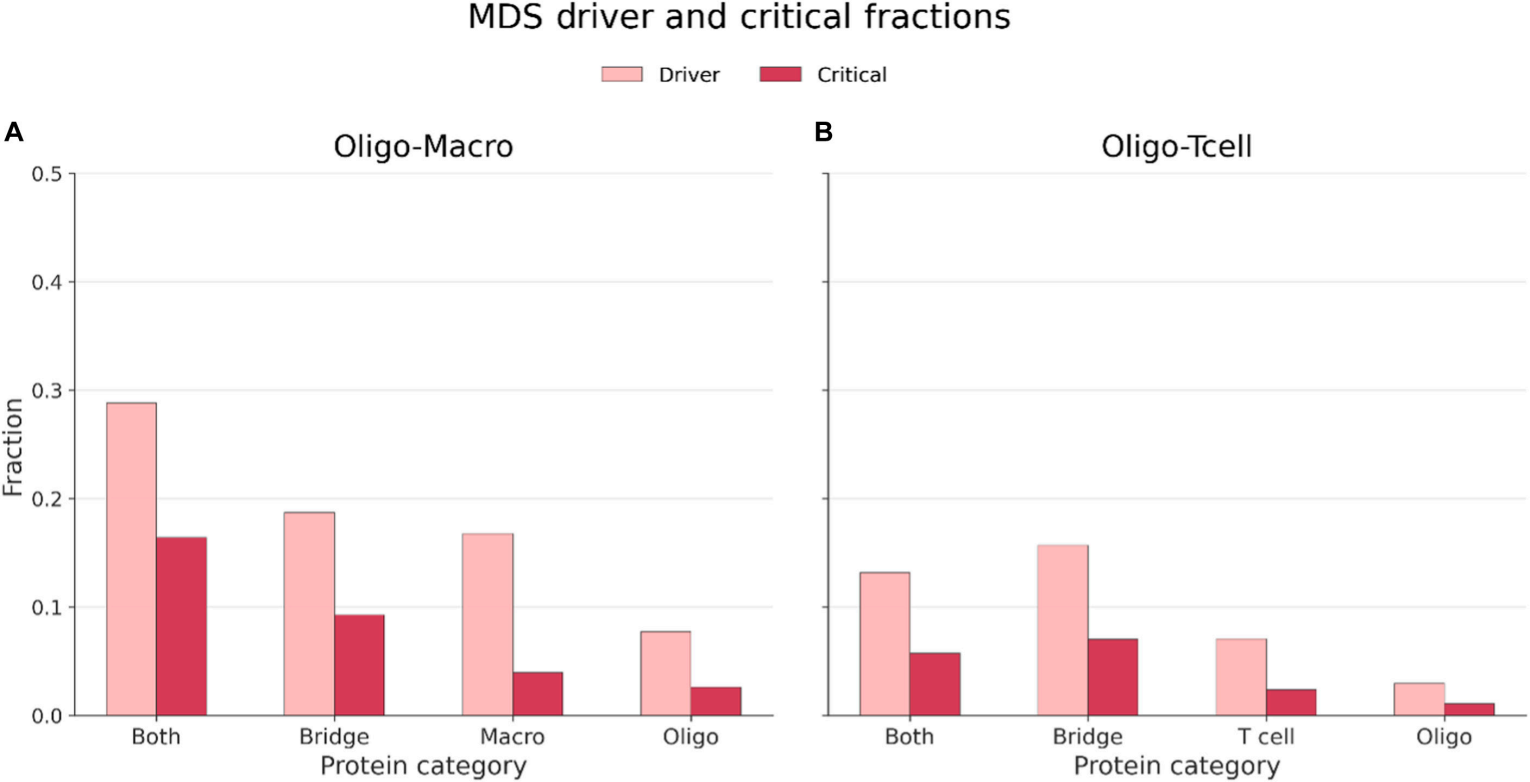
Fraction of MDS driver and critical nodes across proteins categories. Fractions of category nodes identified as MDS control or critical nodes are shown for the (A) OM and (B) OT networks. Protein categories were defined by cell-type assignment: both-cell proteins, bridge proteins mediating ligand–receptor interactions, and cell-type-specific proteins.

We also examined the corresponding localization patterns under the MDS framework (Fig. 7). In contrast to MDO, MDS control and critical nodes were more strongly represented in the both-cell and bridge categories, particularly in the OM network, and in the bridge and both-cell categories in the OT network. This suggests that MDS, which is based on local neighborhood domination in the undirected network, tends to select control-related nodes from shared or intercellular connector categories, whereas MDO identifies control-related nodes according to directed path–cycle cover structures.

Overall, these localization patterns suggest a model-dependent difference: MDS tends to select control-related nodes from shared or bridge-associated protein categories, whereas MDO more strongly highlights cell-type-specific protein categories.

### Enrichment of control-related nodes across protein categories

**Fig. 8.**
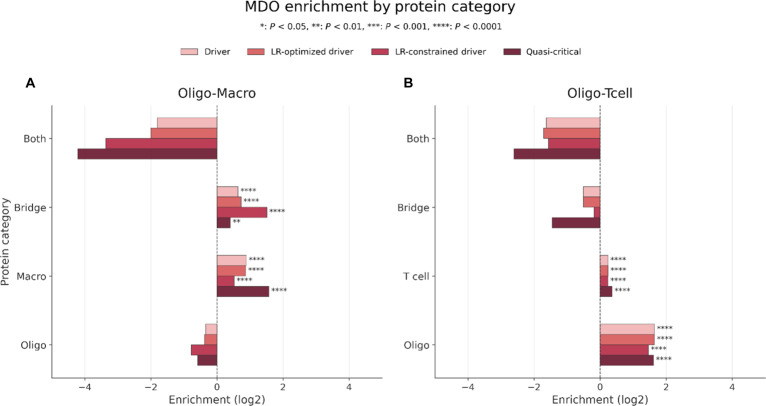
Enrichment of MDO control-related nodes across protein categories. Enrichment analysis of MDO driver and quasi-critical nodes across for protein categories defined by cell-type assignment in the (A) OM and (B) OT networks, with network sizes of *N* = 3,562 and *N* = 8,150 nodes, respectively. Bars represent the log_2_ (fold enrichment) values for each protein category. *P* values were computed using a one-sided Fisher’s exact test to assess overrepresentation. Statistical significance is indicated as follows: *P < 0.05, **P < 0.01, ***P < 0.001, and ****P < 0.0001. Significant positive enrichment was observed in the bridge and macrophage protein categories in OM and in the oligodendrocyte and T-cell categories in OT.

In the previous section, we examined the fractions of control-related nodes within each protein category. We next evaluated whether these observed fractions reflected statistically significant overrepresentation in specific categories. Under the MDO framework, the OM and OT networks showed distinct enrichment patterns (Fig. 8). In the OM network, MDO driver nodes were significantly enriched in the bridge and macrophage protein categories, whereas both-cell proteins showed negative enrichment. This trend was broadly preserved for LR-optimized MDO, while LR-constrained MDO showed particularly strong enrichment in the bridge category. Quasi-critical nodes were most strongly enriched in the macrophage category, followed by weaker enrichment in the bridge category. In contrast, in the OT network, MDO driver and quasi-critical nodes were significantly enriched in the oligodendrocyte and T-cell categories, with the strongest enrichment observed in the oligodendrocyte category. These results indicate that MDO-based control-related nodes are preferentially associated with bridge/macrophage proteins in OM, but with cell-type-specific oligodendrocyte and T-cell proteins in OT.

**Fig. 9.**
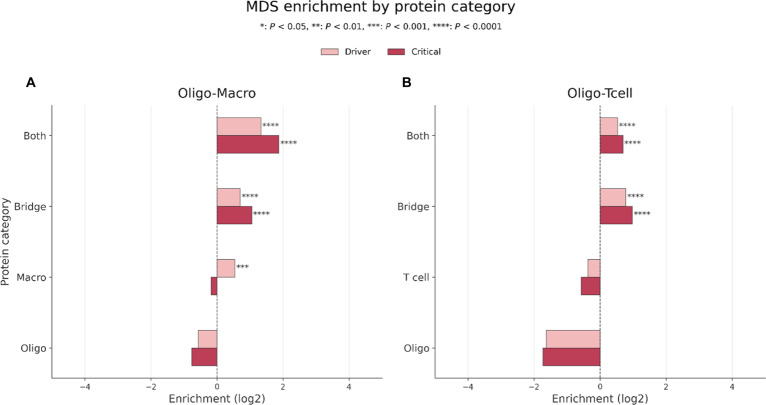
Enrichment of MDS control-related nodes across protein categories. Enrichment analysis of MDS driver and critical nodes across for protein categories defined by cell-type assignment in the (A) OM and (B) OT networks. Bars represent the log_2_ (fold enrichment) values for each protein category. *P* values were computed using a one-sided Fisher’s exact test to assess overrepresentation. Statistical significance is indicated as follows: *P < 0.05, **P < 0.01, ***P < 0.001, and ****P < 0.0001. Significant positive enrichment was observed in the bridge and both protein categories in OM and OT networks.

We then performed the same enrichment analysis under the MDS framework (Fig. 9). In both OM and OT, MDS control and critical nodes were significantly enriched in the both-cell and bridge protein categories. By contrast, cell-type-specific categories showed weaker enrichment or negative enrichment, especially for oligodendrocyte proteins in OT. Thus, compared with MDO, MDS preferentially selected shared or intercellular connector proteins. Overall, these enrichment results support a model-dependent difference in control-node organization: MDO reveals system-dependent enrichment patterns, with bridge/macrophage-associated control in OM and oligodendrocyte/T-cell-associated control in OT, whereas MDS emphasizes shared and bridge-associated protein categories in both systems.

### Network centrality analysis of protein categories

**Fig. 10.**
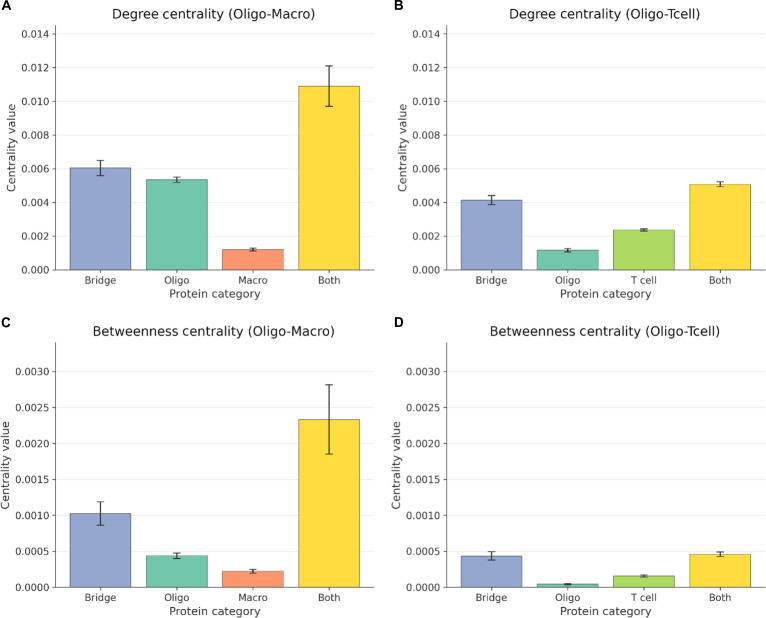
Degree and betweenness centrality across protein categories. Average normalized degree centrality and betweenness centrality are shown for protein categories defined by cell-type assignment in the OM and OT networks. (A and B) Normalized degree centrality in the OM and OT networks. (C and D) Betweenness centrality in the OM and OT networks. Bars indicate mean centrality values, and error bars indicate standard deviation.

To complement the controllability analysis, we next examined the topological properties of proteins in each protein category. Specifically, we compared normalized degree centrality and betweenness centrality across protein categories in the OM and OT interaction networks (Fig. 10). The degree centrality measures how important or central a node is in a network based on the number of connections it has. For a node *i*, it is defined as DC(*i*) *=* deg(*i*)/(*n* − 1), where *n* denotes the total number of nodes in the network and deg(*i*) is the degree of node *i*. On the other hand, the betweenness centrality reflects the extent to which a node lies on shortest paths between other nodes.

Proteins expressed in both cell types showed the highest average degree centrality in both the OM and OT networks (Fig. 10A and B), indicating that these shared proteins tend to occupy highly connected positions. Bridge proteins also showed relatively high degree centrality, particularly in the OT network, consistent with their role in mediating intercellular LR interactions. In contrast, cell-type-specific proteins generally showed lower degree centrality, especially macrophage-specific proteins in OM and oligodendrocyte-specific proteins in OT.

A similar pattern was observed for betweenness centrality (Fig. 10C and D). Proteins expressed in both cell types showed the highest average betweenness centrality in both networks, suggesting that they frequently occupy intermediate positions along network paths. Bridge proteins also displayed relatively high betweenness centrality, supporting their role as intercellular connectors. Together, these results indicate that both-cell and bridge proteins occupy central topological positions in the integrated cell–cell interaction networks.

### Functional enrichment analysis of MDO and MDS-derived control-related nodes

To connect network controllability with biological function, we performed GO enrichment analysis for the driver node sets identified by MDO and MDS. In this analysis, empirical *P* values were computed using 50,000 permutations, and both nominal *P* values from Fisher’s exact test and BH-corrected *q* values are reported in Tables 1 and 2 and Table S1 (see Materials and Methods for details).

In the OM network, MDO driver nodes showed nominal enrichment for several BP, CC, and MF terms, including fibrinolysis, T-cell receptor complex, and carbon–nitrogen ligase activity. However, these terms did not survive BH correction at *q* < 0.10. Therefore, we interpret the MDO-associated GO terms as exploratory functional patterns rather than statistically robust enrichments. We also note that some nominally enriched MDO-OM terms were influenced by closely related fibrinogen-chain proteins, such as FGA, FGB, and FGG. Therefore, these terms should not be interpreted as independent evidence for broad vascular or hemostatic pathway enrichment. Nevertheless, this fibrinogen-complex-related nominal signal may be biologically plausible in the context of MS, because fibrinogen/fibrin deposition has been implicated in blood–brain barrier disruption, microglial activation, neuroinflammation, and demyelinating pathology [29,30]. By contrast, several MDS-associated terms in the OM network remained significant after BH correction, including positive regulation of intracellular signal transduction (BP; GO:1902533; *q* = 1.99 × 10^−4^) and positive regulation of protein phosphorylation (BP; GO:0001934; *q* = 5.44 × 10^−4^). Other terms, such as protein kinase binding (MF; GO:0019901), showed nominal enrichment but did not survive BH correction and were therefore interpreted as exploratory. Full nominal *P* values, empirical *P* values, and BH-corrected *q* values are reported in Table 1 and Table S1.

In the OT network, MDO driver nodes showed nominal enrichment for metabolic, ion-transport, and synapse-associated terms, including nucleotide-sugar metabolic process (BP; GO:0009225), inorganic cation import across plasma membrane (BP; GO:0098659), excitatory synapse (CC; GO:0060076), and potassium:chloride symporter activity (MF; GO:0015379). However, these terms did not survive BH correction at *q* < 0.10. Thus, as in the OM network, the MDO-associated GO results in OT were interpreted cautiously as exploratory functional patterns. In contrast, several MDS-associated terms in the OT network remained significant after BH correction. These included regulation of ERK1 and ERK2 cascade (BP; GO:0070372; *q* = 1.55 × 10^−2^), ubiquitin protein ligase binding (MF; GO:0031625; *q* = 5.37 × 10^−3^), and ubiquitin-like protein ligase binding (MF; GO:0044389; *q* = 7.01 × 10^−3^). In addition, ephrin receptor binding (MF; GO:0046875; *q* = 2.9 × 10^−4^) was strongly enriched among MDS critical nodes and included signaling proteins such as FYN, SRC, GRB2, CRK, PTPN1, NCK1, and EFNB1, suggesting that MDS-critical nodes may capture functionally relevant signaling components in the OT network.

Because LR-optimized MDO preserved a highly similar set of driver nodes to unconstrained MDO (see Fig. S1) as well as the identical set of quasi-critical nodes, its GO-ranking patterns were broadly comparable to those of unconstrained MDO. In contrast, LR-constrained MDO increases the total number of driver nodes, which also altered a fraction of driver-node identities because known LR directions are imposed as hard constraints. Therefore, we treat LR-constrained GO enrichment as a sensitivity analysis. Full GO results for unconstrained MDO, LR-optimized MDO, LR-constrained MDO, MDS driver nodes, and quasi-critical/critical node sets are provided in Table S1 for comparison.

### Comparison with known MS-associated genes

To evaluate whether the control-related node sets recovered previously reported MS-associated proteins, we compared them with a reference set of MS-associated proteins obtained from MSGD (see Materials and Methods and Table 3). In the OM network, MDO driver nodes were significantly enriched for MSGD proteins (enrichment factor = 1.78; *P* = 5.62 × 10^−3^; *P*_emp_ = 5.70 × 10^−3^). The LR-optimized MDO driver set showed essentially the same enrichment, consistent with the high overlap in driver-node identities between unconstrained MDO and LR-optimized MDO (see Fig. S3). In contrast, LR-constrained MDO showed weaker and nonsignificant enrichment for MSGD proteins (enrichment factor = 1.29; *P* = 0.165; *P*_emp_ = 0.167), consistent with the changes in driver-node identities introduced by imposing known LR directions as hard constraints (see Fig. S3). MDO quasi-critical nodes in the OM network showed a weak trend toward enrichment for MSGD proteins but did not reach nominal significance at *P* < 0.05 (enrichment factor = 1.61; *P* = 6.35 × 10^−2^; *P*_emp_ = 6.38 × 10^−2^). In contrast, the MDS framework showed stronger and more consistent recovery of known MS-associated proteins. In the OM network, both MDS driver nodes and MDS critical nodes were enriched for MSGD proteins, with the strongest enrichment observed for MDS driver nodes (enrichment factor = 2.33; *P* = 2.70 × 10^−4^; *P*_emp_ = 2.00 × 10^−4^).

**Table 3.** Enrichment of MSGD multiple-sclerosis-associated proteins among control-related node sets. Enrichment of the human MSGD proteins was evaluated for MDO driver nodes, LR-optimized MDO driver nodes, LR-constrained MDO driver nodes, MDO quasi-critical nodes, MDS driver nodes, and MDS critical nodes in the OM and OT networks. *P* values were computed using a one-sided Fisher’s exact test, and empirical *P* values (*P*_emp_) were obtained from 50,000 permutations. Full overlap lists are provided in Table S2.

Method	Network	Node set	Enrichment factor	*P*_emp_	*P* value
MDO	OM	Driver	1.78	5.70 × 10^−3^	5.62 × 10^−3^
MDO LR optimized	OM	Driver	1.78	5.70 × 10^−3^	5.62 × 10^−3^
MDO LR-constrained	OM	Driver	1.29	1.67 × 10^−1^	1.65 × 10^−1^
MDO	OM	Quasi-critical	1.61	6.38 × 10^−2^	6.35 × 10^−2^
MDS	OM	Driver	2.33	2.00 × 10^−4^	2.70 × 10^−4^
MDS	OM	Critical	2.21	3.71 × 10^−2^	3.74 × 10^−2^
MDO	OT	Driver	0.827	7.36 × 10^−1^	7.33 × 10^−1^
MDO LR-optimized	OT	Driver	0.827	7.36 × 10^−1^	7.33 × 10^−1^
MDO LR-constrained	OT	Driver	0.637	8.94 × 10^−1^	8.84 × 10^−1^
MDO	OT	Quasi-critical	0.796	7.50 × 10^−1^	7.49 × 10^−1^
MDS	OT	Driver	1.99	9.70 × 10^−3^	9.22 × 10^−3^
MDS	OT	Critical	3.29	1.40 × 10^−3^	1.56 × 10^−3^

In the OT network, MDO driver nodes and LR-optimized MDO driver nodes did not show enrichment for MSGD proteins (enrichment factor = 0.827; *P* = 0.733; *P*_emp_ = 0.736), and LR-constrained MDO also remained nonsignificant. Therefore, we do not interpret the lack of MSGD enrichment in the OT-MDO driver set as evidence for novel MS candidates. Instead, this result indicates that the OT-MDO driver set is structurally distinct from the current MSGD protein-evidence reference set. By contrast, MDS control and critical nodes were significantly enriched for MSGD proteins in the OT network, with the strongest enrichment observed for MDS critical nodes (enrichment factor = 3.29; *P* = 1.56 × 10^−3^; *P*_emp_ = 1.40 × 10^−3^).

Regarding the overlapping MS-associated proteins in the OM network, the MDO driver-node overlap with MSGD included proteins representing several MS-relevant biological categories. These included myelin- and oligodendrocyte-associated proteins such as PLP1 and MBP, the oligodendrocyte-lineage transcription factor OLIG2, and immune/inflammatory regulation-related proteins such as CIITA, CCR2, and IFNAR1. These categories are consistent with major aspects of MS biology, including oligodendrocyte/myelin pathology and immune-inflammatory regulation [27,31,32]. These proteins were also retained in the LR-optimized MDO driver set, consistent with the high overlap between unconstrained MDO and LR-optimized MDO driver nodes. Among these highlighted proteins, MBP, CIITA, IFNAR1, and OLIG2 were also present in the MDO quasi-critical node set. The full list of overlapping proteins for all MDO, LR-optimized MDO, LR-constrained MDO, MDS control, and critical/quasi-critical node sets is provided in Table S2. The overlap of driver-node sets and MSGD-associated proteins is visualized using Venn diagrams in Fig. S4.

## Discussion

We introduced MDO, a computational framework that assigns directions to edges in undirected PPI networks so that the number of driver nodes is minimized. This enables the application of MM-based structural controllability analysis to undirected protein interaction networks, including integrated cell–cell interaction networks, which could not previously be analyzed directly in this framework. When applied to OM and OT systems, MDO suggested distinct control-node patterns: the OM network showed a macrophage- and bridge-associated organization, whereas the OT network exhibited a more cell-type-specific control pattern involving oligodendrocyte and T-cell protein categories. From a theoretical perspective, our results showed that MDO establishes the absence of critical nodes in connected graphs and introduces quasi-critical nodes as a distinct category in structural controllability. These results suggest that MDO provides not only a practical method for orienting undirected interaction networks but also a theoretical extension of MM-based controllability to graph settings where edge directions are not known a priori.

We also compared the biological associations obtained from MDO and the established MDS-based controllability framework. In the GO enrichment analysis, MDO-derived driver nodes showed several nominally enriched functional patterns, but these terms did not survive BH correction at *q* < 0.10 and were therefore interpreted as exploratory. In contrast, several MDS-derived driver and critical node sets showed GO enrichments that remained significant after BH correction, suggesting that MDS provided more statistically robust functional signals in the present dataset. Similarly, enrichment for known MSGD multiple-sclerosis-associated proteins was observed for MDS-derived node sets in both OM and OT networks, whereas MDO-derived driver nodes showed significant MSGD enrichment only in the OM network. Thus, the analysis indicates that MDS more consistently recovers known functional and MS-associated annotations, whereas MDO captures a different aspect of control organization based on directed path–cycle cover structures.

Importantly, MDO should not be interpreted as a direct predictor of biological LR directionality. Consistent with the path–cycle cover formulation and the theoretical results in Theorem 1 and Proposition 1, MDO primarily determines the underlying path–cycle cover structure, whereas some edge orientations can remain ambiguous and may require additional information. Consistent with this interpretation, LR-optimized MDO improved agreement with known LR directions by selecting among MDO-optimal orientations, while preserving the same minimum driver-node count as unconstrained MDO. Thus, LR information can be incorporated as an additional criterion without changing the controllability optimum. In contrast, LR-constrained MDO imposed known LR directions as hard constraints and therefore changed the optimization problem; although the driver-node fraction remained close to that of unconstrained MDO, driver-node identities and associated enrichment results were altered. Therefore, LR-constrained MDO was treated as a sensitivity analysis rather than as a replacement for the unconstrained or LR-optimized MDO models. Overall, MDO-derived edge directions should be interpreted as controllability-optimized orientations, whereas the main biological interpretation should focus on the selected driver nodes, quasi-critical nodes, and their localization across protein categories.

It is also noteworthy that the quasi-critical node set was unchanged across unconstrained MDO, LR-optimized MDO, and LR-constrained MDO approaches. Although MDO quasi-critical nodes showed limited overlap with currently annotated MSGD proteins, this may partly reflect their peripheral or low-degree character, since such proteins are generally less extensively studied than major hubs or highly connected proteins, which are more likely to be emphasized by local neighborhood-based control models such as MDS. From the MDO perspective, however, quasi-critical nodes remain topologically important because they can become driver nodes under MDO-optimal orientations and may represent latent control-entry points in the directed path–cycle cover structure.

It is also notable that, in the larger OT network, MDO required fewer driver nodes than MDS, suggesting that the MM-based framework can provide a compact control representation in larger interaction systems. However, because MDO and MDS are based on different notions of control, this difference should not be interpreted as evidence that one model is universally superior. Instead, MDO and MDS should be regarded as complementary approaches: MDO captures control organization through optimized directed path–cycle covers, whereas MDS captures local neighborhood-based domination in the undirected network.

Several limitations should be noted. First, the present study is entirely computational and is based on previously published cell–cell interaction datasets; therefore, the identified control-node patterns should be interpreted as network-derived hypotheses rather than experimentally validated biological mechanisms. Second, the GO enrichment and MSGD overlap analyses provide exploratory biological annotation rather than independent functional validation, especially for MDO-derived terms that did not survive BH correction. Complementary pathway analyses using resources such as Reactome or MSigDB Hallmark, as well as validation using transcriptomic or single-cell RNA-seq datasets from MS lesions, would be valuable future directions.

Beyond these biological validation issues, the proposed MDO framework could be extended to other large-scale undirected cell–cell interaction networks, such as tumor–immune or neuron–glia systems, provided that intracellular interaction networks for the relevant cell types can be integrated with reliable intercellular signaling interactions. Another important direction is the analysis of multicellular systems involving more than 2 interacting cell types, which may reveal higher-order control structures that cannot be captured in pairwise cell–cell networks. Finally, improving the scalability of MDO-based analyses for larger multicellular systems remains an important topic for future research. Work is currently underway to develop an algorithmic extension that does not rely on ILP and that can more efficiently identify control-related nodes in large-scale cell–cell interaction networks.

## Data Availability

The datasets analyzed in this study were obtained from previously published sources and public databases cited in the article. The main MDO Control code used in this study is available at GitHub (https://github.com/NacxLab/ControlMDO/). Additional data supporting the findings of this study are available from the corresponding author upon reasonable request.
