# Supplementary material for: Controllability Analysis of Intercellular Protein–Protein Interaction Networks
Source: Comput Struct Biotechnol J. 2026 Aug 18;35(1):0177. doi: 10.34133/csbj.0177 (PMC13481791; doi:10.34133/csbj.0177)
Supplement: Supplementary file 1 [file csbj.0177.f1.zip › Supplementary Material_Figs_S1_S2_S3_S4.pdf]

Supplementary Figures

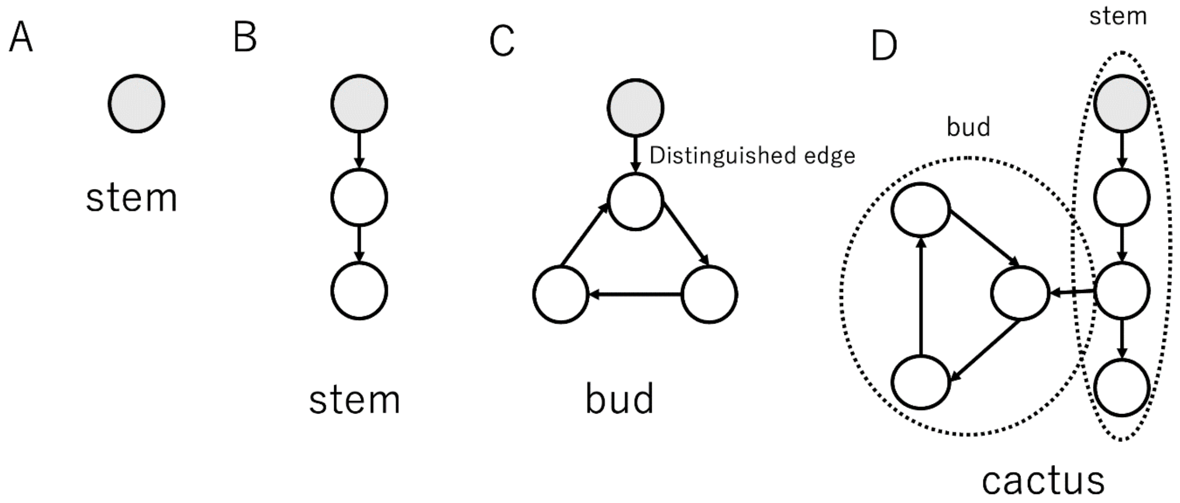

Supplementary Fig. S1. Examples of stems, buds, and cactus structures. (A-B) Examples of stems, including an isolated node regarded as a stem of length zero and a simple directed path, defines as a directed path that does not pass through the same node more than once. (C) Example of a bud, consisting of a directed cycle and a distinguished edge entering the cycle from an outside node. (D) Example of a cactus formed by attaching a bud to an existing stem. Dotted outlines indicate the stem and bud components.

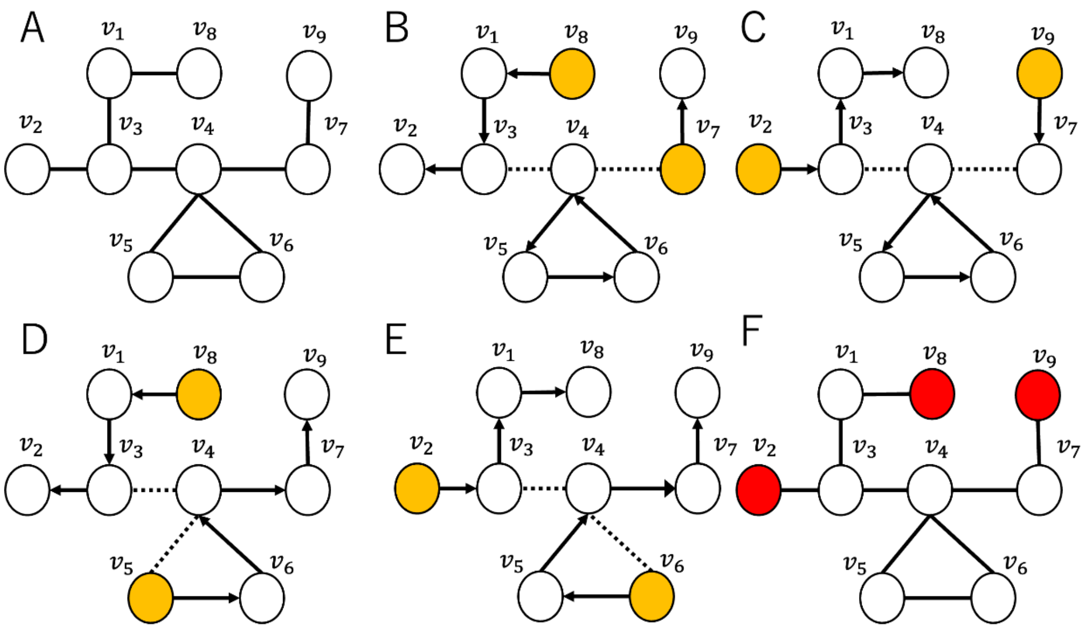

Supplementary Fig. S2. Example of multiple MDO-optimal orientations.
(A) Original undirected graph. (B-E) Different MDO-optimal orientations/path–cycle covers obtained from the same undirected graph. Orange nodes indicate driver nodes. Solid arrows indicate directed edges included in the corresponding MDO solution, whereas dotted lines indicate edges from the original graph that are not used in the selected path–cycle cover. The examples show that different MDO-optimal orientations can have the same minimum number of driver nodes but different driver-node identities. (F) Highlighted nodes in red have degree 1 in the underlying undirected graph and are therefore quasi-critical nodes by Proposition 1. This means they appear as endpoints of paths in every optimal path–cycle cover, although they do not necessarily become driver nodes in every MDO-optimal orientation.

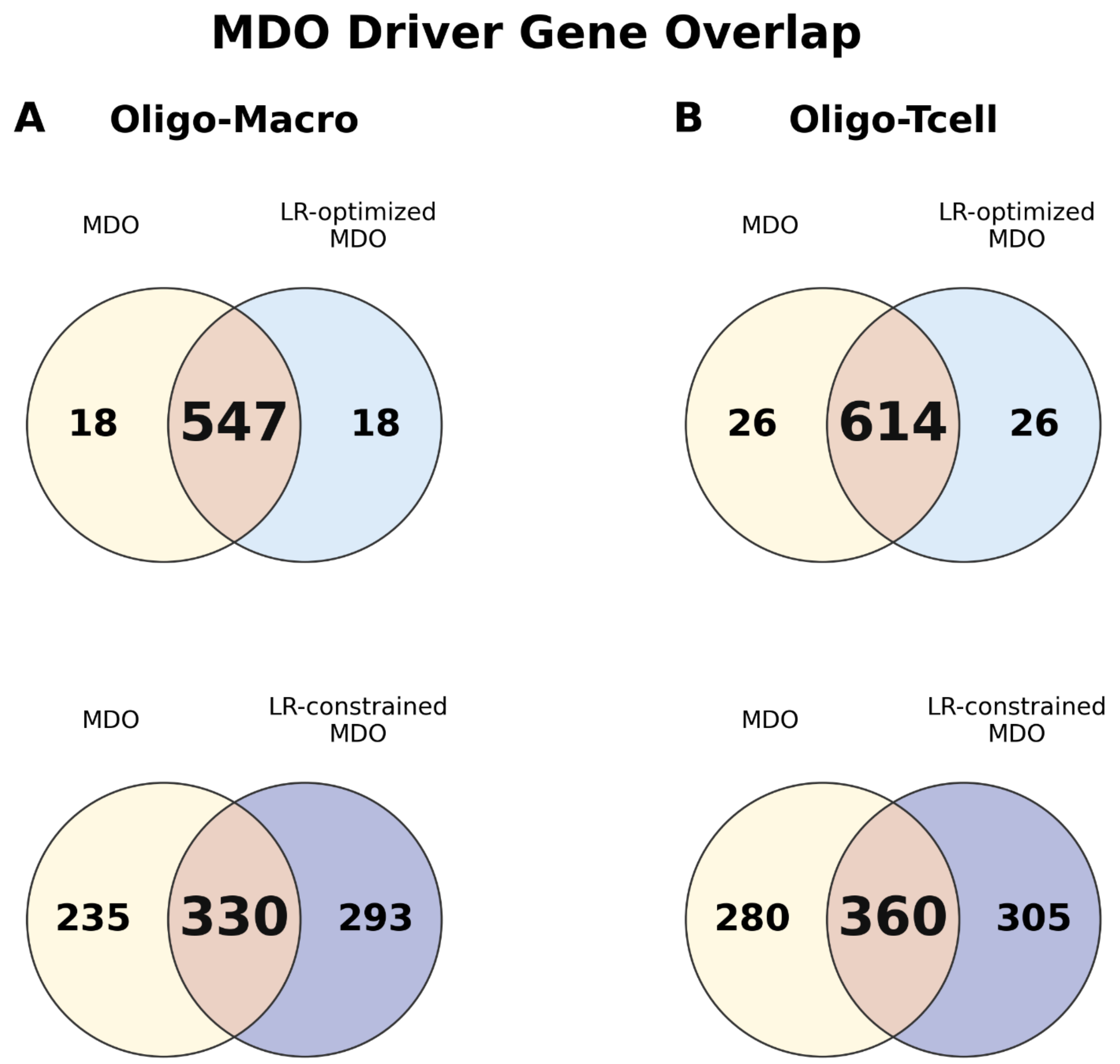

Supplementary Fig. S3. Overlap of MDO driver-node sets across LR-based MDO variants. Venn diagrams show the overlap between unconstrained MDO and LR-optimized MDO, and between unconstrained MDO and LR-constrained MDO, in the (A) OM and (B) OT networks. Numbers indicate shared and method-specific driver nodes. The quasi-critical node set was identical across the MDO variants.

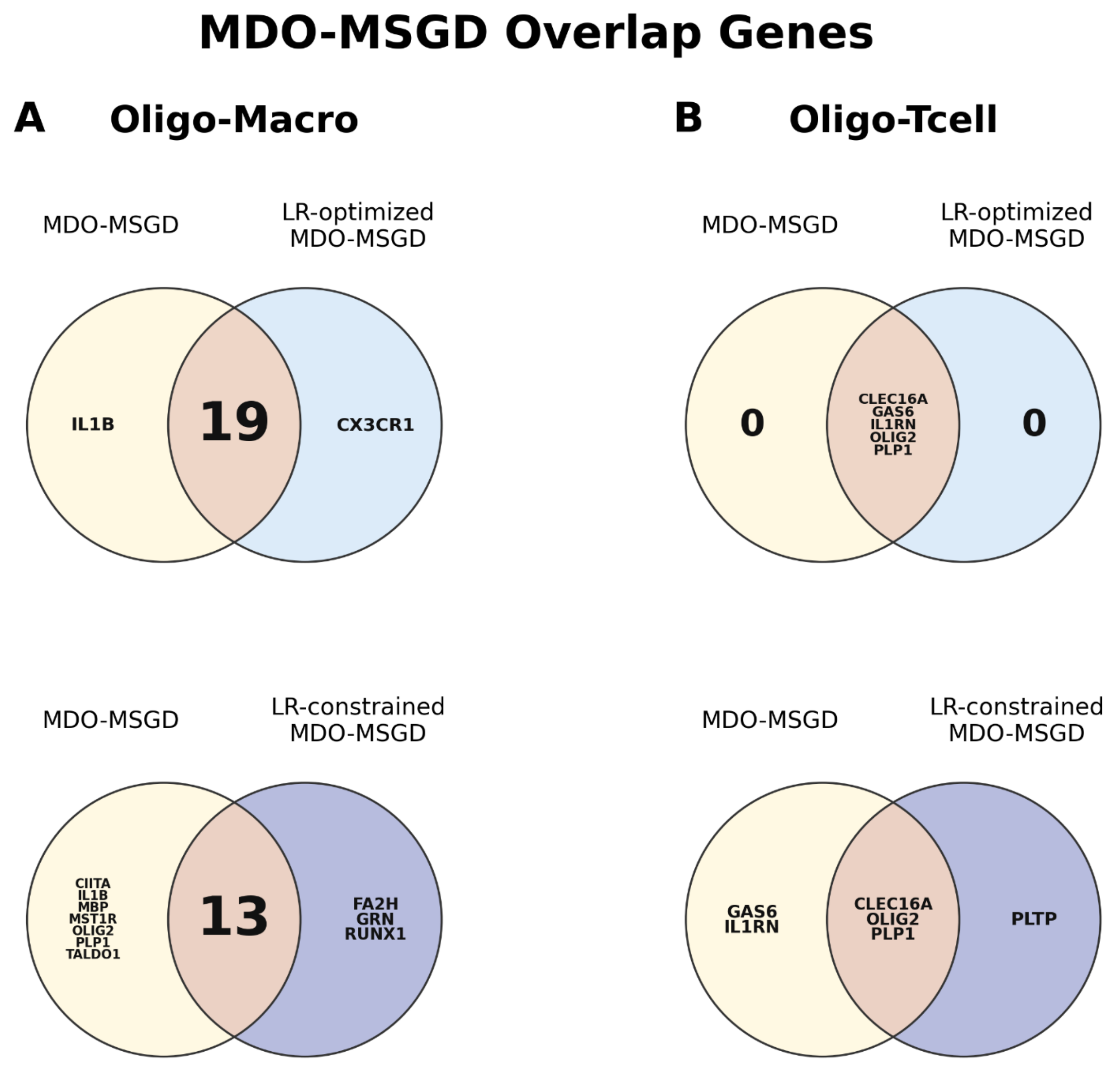

Supplementary Fig. S4. Overlap of MSGD-associated proteins among MDO driver-node sets. Venn diagrams show MSGD-associated proteins overlapping with driver-node sets obtained from unconstrained MDO, LR-optimized MDO, and LR-constrained MDO in the (A) OM and (B) OT networks. For each network, overlaps are shown between unconstrained MDO and LR-optimized MDO, and between unconstrained MDO and LR-constrained MDO. Gene names indicate shared and method-specific MSGD-overlapping proteins. Full overlap lists are provided in Supplementary Table S2.
